# MPT0B169, a New Antitubulin Agent, Inhibits Bcr-Abl Expression and Induces Mitochondrion-Mediated Apoptosis in Nonresistant and Imatinib-Resistant Chronic Myeloid Leukemia Cells

**DOI:** 10.1371/journal.pone.0148093

**Published:** 2016-01-27

**Authors:** Shuit-Mun Wong, Fu-Hwa Liu, Yueh-Lun Lee, Huei-Mei Huang

**Affiliations:** 1 Graduate Institute of Medical Sciences, College of Medicine, Taipei Medical University, Taipei, Taiwan; 2 Institute of Molecular Biology, Academia Sinica, Taipei, Taiwan; 3 Department of Microbiology and Immunology, College of Medicine, Taipei Medical University, Taipei, Taiwan; Università degli Studi di Firenze, ITALY

## Abstract

Chronic myeloid leukemia (CML) is a clonal disorder of hematopoietic stem/progenitor cells that is caused by the Bcr-Abl oncoprotein. Clinical resistance to the Bcr-Abl inhibitor imatinib is a critical problem in treating CML. This study investigated the antitumor effect and mechanism of MPT0B169, a new antitubulin agent, in K562 CML cells and their derived imatinib-resistant cells, IMR2 and IMR3. IMR2 and IMR3 cells showed complete resistance to imatinib-induced growth inhibition and apoptosis. Resistance involved ERK1/2 overactivation and MDR1 overexpression. MPT0B169 inhibited the growth of K562, IMR2, and IMR3 cells in a dose- and time-dependent manner. MPT0B169 substantially inhibited the mRNA and protein levels of Bcr-Abl, followed by its downstream pathways including Akt, ERK1/2, and STAT3 in these cells. MPT0B169 treatment resulted in a decrease in the polymer form of tubulin according to Western blot analysis. It triggered cell cycle arrest at the G2/M phase before apoptosis, which was related to the upregulation of the mitotic marker MPM2 and the cyclin B1 level, and a change in the phosphorylation of Cdk1. MPT0B169 induced apoptosis in nonresistant and imatinib-resistant cells via a mitochondrion-mediated caspase pathway. Further study showed that the agent led to a decrease in the antiapoptotic proteins Bcl-2, Bcl-xL, and Mcl-1 and an increase in the apoptotic protein Bax. Taken together, our results suggest that MPT0B169 might be a promising agent for overcoming imatinib resistance in CML cells.

## Introduction

Chronic myeloid leukemia (CML) is a malignant disorder of hematopoietic stem/progenitor cells characterized by the reciprocal translocation between chromosomes 9 and 22 t(9;22) leading to the formation of the Philadelphia (Ph) chromosome [1]. Bcr-Abl protein, a constitutively activated tyrosine kinase, is the product of the chimeric *Bcr-Abl* fusion gene on the Ph chromosome [1]. Bcr-Abl constitutively activates downstream effector pathways that stimulate cell proliferation and protect cells from apoptosis, such as Akt, ERK1/2, and STAT3 [2,3].

Imatinib (STI571, Gleevec), a Bcr-Abl tyrosine kinase inhibitor, is highly effective and is currently the first-line therapy for CML [4]. In addition, several first-line drugs are available for therapeutic use in CML, including nilotinib and dasatinib [5–7]. Although imatinib has improved clinical outcomes in the chronic phase of CML, drug resistance emerged in some patients, especially in the accelerated phase and blast crisis. Second- and third-generation inhibitors are effective against most imatinib-resistant (IMR) CML, but some patients become resistant to these drugs [8]. Hence, there is still an urgent need to develop novel agents that can be used to overcome Bcr-Abl inhibitor resistance.

Microtubules are cytoskeletal fibers consisting of polymerized heterodimers of α- and β-tubulin, which play crucial roles in maintaining cell growth, cell shape, and cell–cell interactions. Cancer cells exhibit a strong growth rate and they require microtubules to undergo division [9]. Therefore, tubulin is one of the most attractive targets of anticancer approaches. Recently, antitubulin agents targeting the colchicine-binding site of tubulin have become promising anticancer drugs, some of which have entered clinical trials [10].

**Fig 1:**
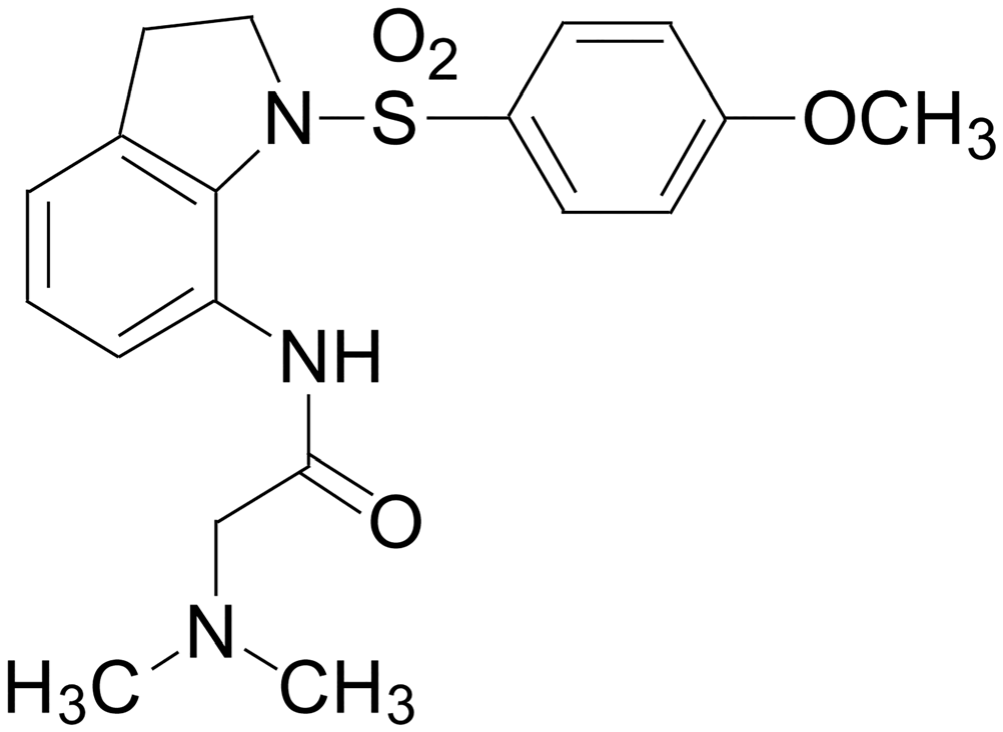
Chemical structure of MPT0B169.

We previously synthesized a novel tubulin inhibitor, MPT0B169 (2-dimethylamino-N-[1-(4-methoxy-benzenesulfonyl)-2,3-dihydro-1H-indol-7-yl]-acetamide) (Fig 1), which binds to the colchicine binding site of tubulin and inhibits microtubule assembly and cell proliferation in acute myeloid leukemia (AML) cells [11]. In this study, we generated IMR clones from K562 CML cells. We studied whether MPT0B169 affects Bcr-Abl expression and its signaling in these cells. The effects of MPT0B169 on tubulin polymerization, the cell cycle, cell growth, and apoptosis in nonresistant and IMR CML cells were also investigated.

## Materials and Methods

### Reagents and antibodies

Imatinib was provided by Novartis Pharma AG (Basel, Switzerland). Antibodies for Western blotting, including caspase-9, caspase-3, cleaved caspase-3, PARP, phospho-c-Abl, phospho-Elk-1, phospho-cyclin-dependent kinase 1 (Cdk1) (Thr161), phospho-Cdk1 (Tyr15), phospho-ERK1/2, ERK1/2, phospho-Akt, Akt, phospho-STAT3, STAT3, Bcl-2, and Bcl-xL, were purchased from Cell Signaling Technology (Danvers, MA, USA). Antibodies specific for c-Abl, multidrug resistance 1 (MDR1), α-tubulin, cyclin B1, Cdk1, Mcl-1, Bax, cytochrome c, and β-actin were purchased from Santa Cruz Biotechnology (Santa Cruz, CA, USA). An antiphosphospecific MPM2 monoclonal antibody was purchased from Upstate Biotechnology (Lake Placid, NY, USA).

### Cell lines

The K562 human CML blast crisis cell line was purchased from the Bioresource Collection and Research Center (BCRC), Hsin-Chu, Taiwan (BCRC 60007) and cultured in RPMI 1640 medium supplemented with 10% fetal bovine serum, 2 mM L-glutamine, 100 units/mL penicillin, and 100 μg/mL streptomycin in a 5% CO_2_ incubator at 37°C.

### Generation of IMR cell clones

IMR clones were derived from K562 cells by exposing them to increasing concentrations of imatinib starting from 100 nM. The concentration of imatinib was doubled every week. After 2 months, cells were cultured in the presence of 10 μM imatinib. These mixed clones were then diluted at 0.5 cell/well in 96-well plates. After 2 weeks of culture, we randomly selected three different clones (IMR1, IMR2, and IMR3).

### Cell proliferation assay

An MTT assay was performed to assess cell viability. K562 and IMR cells were treated with imatinib or MPT0B169 for the indicated time points, and the MTT assay was conducted as described previously [11].

### Soft agarose assay

The colony-forming activity of CML cells was analyzed using a soft agarose assay. Base layers consisting of RPMI 1640 growth medium and 0.6% agarose were added to 6-well plates. K562, IMR2, and IMR3 cells were plated at a density of 2500 cells/well in top layers consisting of RPMI 1640 growth medium and 0.3% agarose and allowed to grow for 2–3 weeks at 37°C. Tumor cell colonies were counted after staining with crystal violet.

### Assessment of cell apoptosis

Apoptosis was analyzed by two methods: a DNA fragmentation assay and annexin V/propidium iodide (PI) staining.

For the DNA fragmentation assay, cells were lysed in ladder assay buffer (10 mM Tris-HCl at pH 8.0, 1 mM EDTA, 0.1% SDS, 0.2 mg/mL RNase, and 0.5 mg/mL proteinase K) and incubated at 37°C for 12 h. Total DNA was extracted using the conventional phenol/chloroform method and isolated using the isopropanol precipitation method. Equal amounts of DNA were separated through 1.5% agarose gel electrophoresis.

The rate of cell apoptosis was quantified by annexin V-FITC and PI staining [12]. Cells were stained with annexin V-FITC and PI (Invitrogen, Carlsbad, CA, USA) and incubated for 15 min at room temperature in the dark. Samples of 10^4^ cells for each scan were acquired on a FACScan flow cytometer (Becton Dickinson, Mountain View, CA, USA) and analyzed using Cellquest software (Becton Dickinson). Results are shown as the percentage of early (annexin V^+^ PI^–^) and late (annexin V^+^ PI^+^) apoptotic cells.

### Western blot analysis

Cell extracts were prepared as described previously [13]. Equal amounts of protein were separated using SDS-polyacrylamide gel electrophoresis, transferred to polyvinylidene difluoride membranes (Millipore, Bedford, MA, USA), and blocked with 5% nonfat milk and 1% Tween-20 in a Tris-buffered solution. Membranes were probed with the primary antibody overnight. After incubation with the appropriate horseradish peroxidase-conjugated secondary antibody, blots were detected by enhanced chemiluminescence detection reagents (PerkinElmer, Waltham, MA, USA) and exposed to X-ray film (Super HR, Fujifilm, Japan). Protein levels were quantified using ImageJ software (http://rsbweb.nih.gov/ij/).

### *In vitro* microtubule assembly assay

Cells were lysed in a lysis buffer (20 mmol/L Tris-HCl at pH 6.8, 1 mmol/L MgCl_2_, 2 mmol/L EGTA, 1 mmol/L PMSF, 1 mmol/L orthovanadate, 0.5% NP-40, and 20 μg/mL for each of the following protease inhibitors: aprotinin, leupeptin, and pepstatin). Microtubule polymers (insoluble tubulin fractions) were separated from free tubulin dimers (soluble tubulin fractions) by centrifugation at 12,000 *g* and 4°C for 10 min Equal amounts of the two fractions were analyzed by Western blotting with an antibody against α-tubulin.

### Analysis of cell surface MDR1 expression

Cell surface expression of the MDR1 protein was analyzed by flow cytometry. Cells were washed three times with cold PBS. All subsequent labeling steps were performed on ice. Cells were incubated with an anti-MDR1 antibody for 30 min. Cells were then washed twice in cold PBS and incubated with a FITC-conjugated goat antimouse IgG antibody. After 30 min, cells were washed three times with PBS. The fluorescence intensity of surface-bound antibodies was analyzed using a FACScan flow cytometer.

### *In vitro* protein kinase assay

An ERK1/2 *in vitro* kinase assay was performed using a p44/42 ERK1/2 kinase assay kit (Cell Signaling Technology, Beverly, MA, USA) following the manufacturer’s instructions. Briefly, total cell extracts were prepared as described [13], and ERK1/2 was immunoprecipitated with an immobilized phospho-ERK1/2 monoclonal antibody. After being washed twice with lysis buffer and twice with kinase buffer (25 mM Tris-HCl, pH7.5, 5 mM β-glycerolphosphate, 2 mM DTT, 0.1 mM Na_3_VO_4_, and 10 mM MgCl_2_), the immunoprecipitates were assayed for ERK1/2 activity in kinase buffer with 200 μM ATP and 0.25 μg of Elk-1 fusion protein per reaction. The reaction was stopped with SDS sample buffer and analyzed by Western blotting with a specific antiphospho-Elk-1 antibody.

### Cell cycle analysis

The cell cycle distribution was assessed as described previously [11]. In brief, cells were washed with PBS and fixed in cold 75% ethanol overnight. Fixed cells were then treated with 50 μg/mL RNase A, stained with 20 μg/mL PI, and stored in the dark for 20 min. The DNA content was determined using a FACScan flow cytometer. The cell cycle distribution was calculated using ModFit LT software (Verity Software House, Topsham, ME, USA).

### Caspase enzymatic activity assay

The activity of caspase-9 and caspase-3 was measured using a colorimetric assay kit (Biovision, Mountain View, CA, USA) according to the manufacturer’s instructions. Briefly, cells were lysed, and the caspase substrates were incubated with cell lysates at 37°C for 1 h and measured at a wavelength of 405 nm in a microtiter plate reader.

### Measurement of the mitochondrial membrane potential

Mitochondrial membrane potential (ΔΨ_m_) was measured by staining cells with 5,5’,6,6’-tetrachloro-1,1’,3,3’-tetraethylbenzimidazolyl-arbocyanine iodide (JC-1, Calbiochem, CA, USA). After MPT0B169 treatment, cells were incubated with JC-1 (5 μg/mL) at 37°C for 15 min. Cells were washed twice with ice-cold PBS. The intracellular red (590 nm) and green (525 nm) fluorescence emissions of JC-1 were then analyzed by flow cytometry. JC-1 dye can enter the mitochondria selectively and form red fluorescent J-aggregates. However, the green fluorescent JC-1 occurs as a monomer in cells with a loss of ΔΨ_m_. The percentage of cells that lost ΔΨ_m_ was estimated using CellQuest software (Becton Dickinson).

### Cytochrome c analysis

Cells were treated with MPT0B169, and cytosolic fractions were generated using a cytosol isolation kit (Sigma, St. Louis, MO, USA) according to the manufacturer’s protocol. The cytosolic fraction at 30 μg was then subjected to a Western blot analysis with an anti-cytochrome c antibody.

### RT-PCR and quantitative real-time PCR

Total RNA was extracted using TRIzol reagent (Invitrogen, CA, USA) according to the manufacturer’s protocol. RT-PCR was performed as described previously [14]. The primers used were Bcr-Abl forward 5'-TTCAGAAGCTTCTCCCTGACAT-3’ and reverse 5’-CTTCGTCTGAGATACTGGATTCCT-3’, and β-actin forward 5’-GCATCCCCCAAAGTTCACAA-3’ and reverse 5’-AGGACTGGGCCATTCTCCTT-3’. The cDNA was quantified by quantitative real-time PCR, using primer pairs with the QuantiFast SYBR Green PCR Kit (Qiagen, Hilden, Germany) on a Roter-Gene Q real-time PCR machine (Qiagen). The normalized gene expression was calculated relative to GAPDH (Qiagen, QT00079247).

### Statistical analysis

Quantitative data are presented as the mean and + standard error of the mean (SEM) of at least three sets of independent experiments. Statistically significant differences were analyzed using Student’s *t*-test. A *p* value of < 0.05 was considered significant.

## Results

### Selection and characterization of IMR cell clones

**Fig 2:**
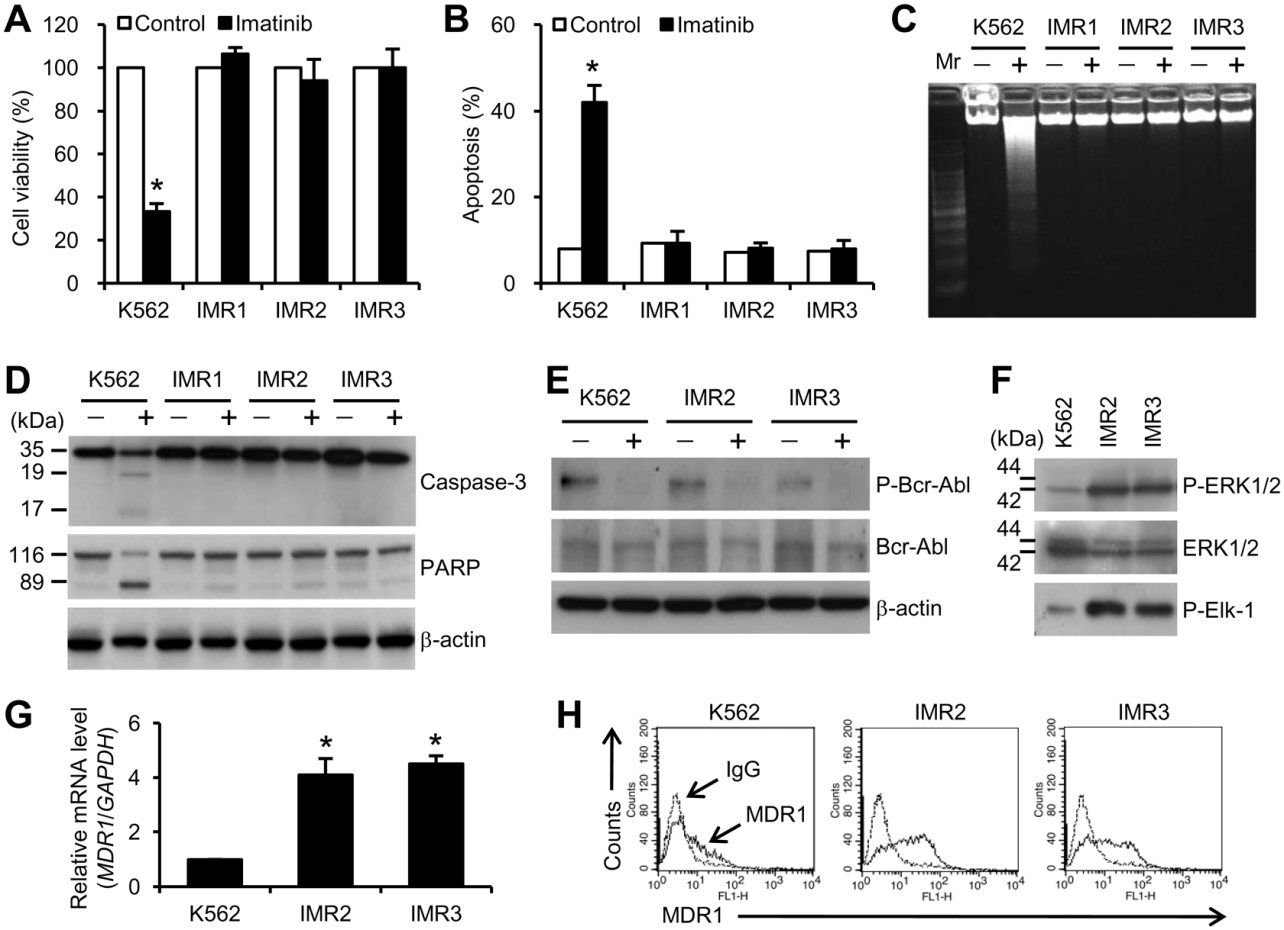
Identification and characterization of IMR clones. K562 and IMR cells were treated with or without 1 μM imatinib for 48 h, and the following experiments were then undertaken. (A) MTT assay. (B) Apoptosis analysis with annexin V/PI staining and flow cytometry. (C) DNA fragmentation assay. (D and E) Western blot analysis was performed with the indicated antibodies. β-actin was used as a loading control. (F) Cell lysates were verified by detecting phospho-ERK1/2 and ERK1/2. The kinase activity of ERK1/2 was determined by an *in vitro* kinase assay with Elk-1 as a substrate. (G) Quantitative real-time PCR showed the overexpression of *MDR1* in IMR2 and IMR3 cells. (H) Flow cytometry showed the levels of MDR1 membrane proteins. A, B, and G show the results of three independent experiments. * *p* < 0.05 versus the untreated control. C to F and H each show one representative result of three independent experiments.

We generated the IMR clones IMR1, IMR2, and IMR3 from K562 cells. We used 1 μM imatinib to conduct experiments; this concentration is clinically relevant and has been previously shown to inhibit the growth of CML cells [15]. All of these IMR clones exhibited strong protection against imatinib-induced growth inhibition (Fig 2A) and apoptosis (Fig 2B) compared with parental K562 cells after 1 μM imatinib treatment. Imatinib did not trigger DNA fragmentation in these IMR clones (Fig 2C). The cleavage of caspase-3 and PARP was also examined. As shown in Fig 2D, the cleavage of caspase-3 (35 kDa) into 19- and 17-kDa products was induced by imatinib concomitantly with that of the caspase-3 substrate, PARP (116 kDa), into an 89-kDa cleaved form in K562 cells, but not in IMR clones. The abilities of IMR1, IMR2, and IMR3 cells to resist imatinib-mediated cytotoxicity were highly similar to each other; we selected IMR2 and IMR3 cells for further study.

To determine whether IMR was associated with an alteration of Bcr-Abl expression, immunoblotting was performed on these cells. As shown in Fig 2E, imatinib inhibited the phosphorylation of Bcr-Abl in K562, IMR2, and IMR3 cells. After imatinib treatment, the protein levels of Bcr-Abl did not change in any of these cells (Fig 2E). An immunoblot analysis to detect the phosphorylation state of total proteins showed the increased phosphorylation of a protein with an approximate molecular weight of 40–50 kDa in IMR2 and IMR3 cells (data not shown). A protein in the 40–50-kDa range likely corresponding to ERK1/2, which is involved in imatinib resistance, was reported [16]. To verify that this protein was ERK1/2, an immunoblot analysis with an antiphospho-ERK1/2 antibody and an *in vitro* kinase assay were performed. Indeed, increases in the phosphorylation level and the kinase activity of ERK1/2 were detected in IMR2 and IMR3 cells (Fig 2F). MDR1 (also known as P-glycoprotein and ATP-binding cassette B1) is a multidrug efflux transporter [17]. MDR1 pumps compounds out of the cancer cells that are associated with multidrug resistance leading to a poor outcome of chemotherapeutic treatment [17]. MDR1 overexpression also contributes to imatinib resistance in CML cells [18]. We subsequently used quantitative real-time PCR and flow cytometry analyses to identify whether the change in MDR1 expression accounted for the resistance to imatinib. Accordingly, mRNA and protein overexpression of MDR1 was observed in IMR2 and IMR3 cells (Fig 2G and 2H). These results suggest that ERK1/2 overactivation and MDR-1 overexpression were associated with resistance against imatinib in IMR2 and IMR3 cells.

### MPT0B169 inhibits cell growth in K562 and IMR cells in a dose- and time-dependent manner

**Fig 3:**
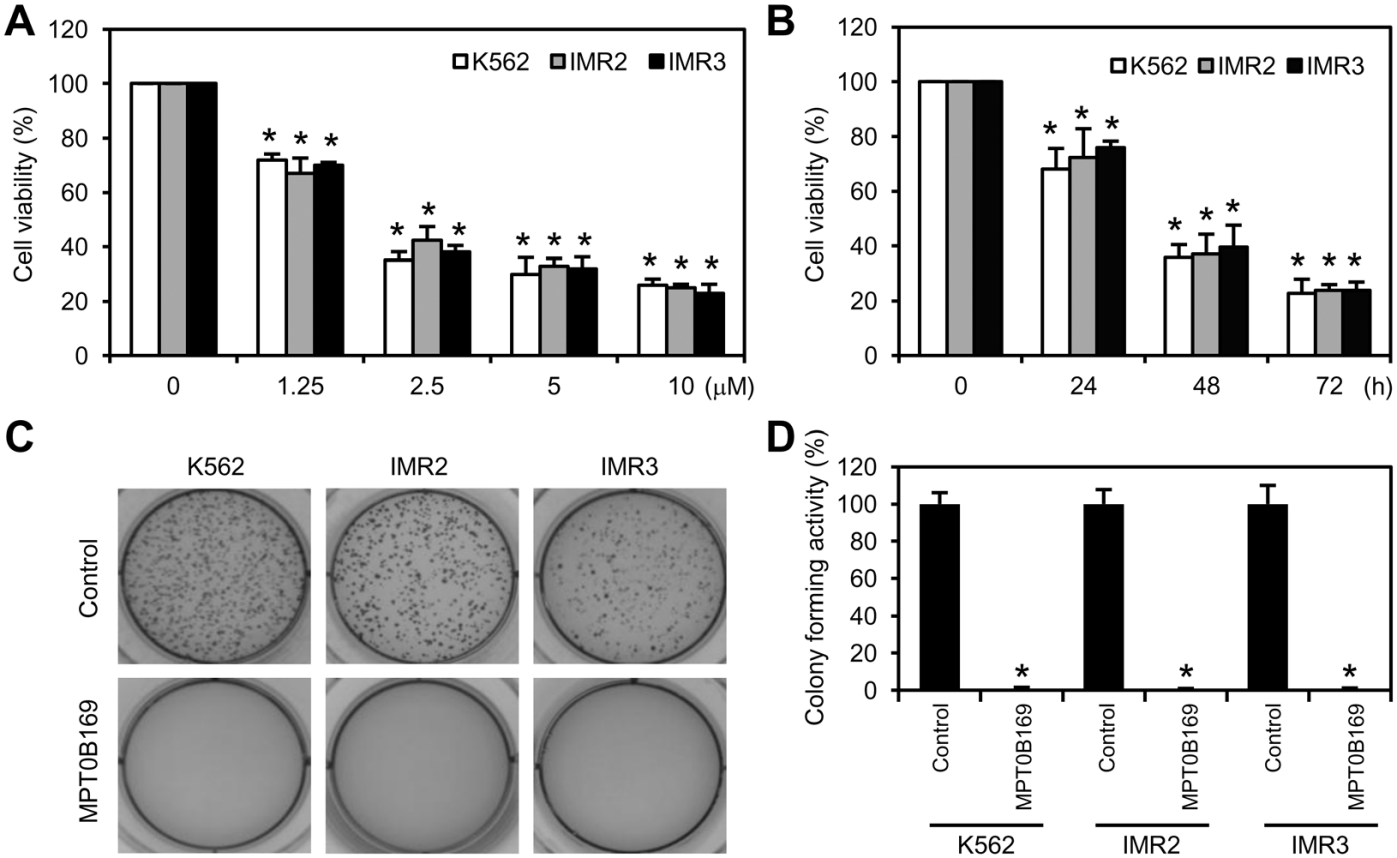
MPT0B169 inhibits the growth of K562, IMR2, and IMR3 cells. (A) Cells were exposed to the indicated concentration of MPT0B169 for 48 h. MPT0B169 inhibited cell viability in a dose-dependent manner according to an MTT assay. (B) Cells were exposed to 2.5 μM MPT0B169 for 0–72 h. MPT0B169 inhibited cell viability in a time-dependent manner according to an MTT assay. (C) MPT0B169 (2.5 μM) inhibited the colony formation of K562, IMR2, and IMR3 cells. These data show representative results from one of three independent experiments. (D) The percentage analysis of colony-forming activity shown in (C). Values are the mean ± SEM relative to the untreated control from three independent experiments. * *p* < 0.05 versus the untreated control (A, B, and D).

To investigate the effect of MPT0B169 on the growth of K562, IMR2, and IMR3 cells, cells were treated with different concentrations of MPT0B169 (1.25–10 μM) for 48 h and an MTT assay was performed. As shown in Fig 3A, MPT0B169 inhibited the viability of K562, IMR2, and IMR3 cells in a dose-dependent manner. We then used 2.5 μM MPT0B169 to analyze the time course of MPT0B169-induced viability reduction and to conduct further study. The viability of K562, IMR2, and IMR3 cells was inhibited by MPT0B169 in a time-dependent manner (Fig 3B). Results of the soft agarose assay demonstrated that MPT0B169 inhibited the colony-forming activity in K562, IMR2, and IMR3 cells, indicating that MPT0B169 inhibited the growth properties of these cells (Fig 3C and 3D).

### MPT0B169 inhibits Bcr-Abl expression and its downstream signaling pathways

**Fig 4:**
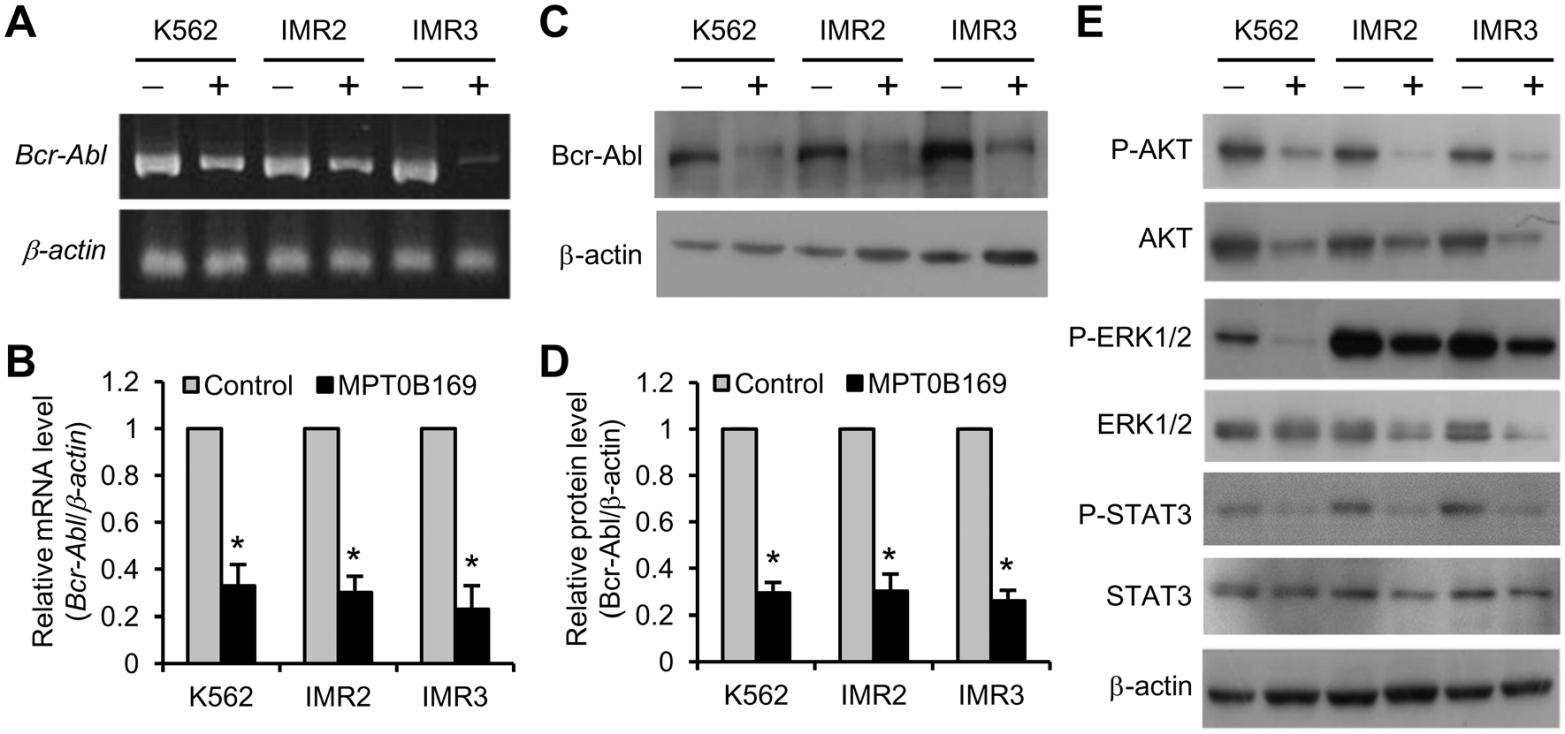
MPT0B169 reduces Bcr-Abl expression and its downstream signaling pathways. Cells were treated with or without 2.5 μM MPT0B169 for 48 h, and the following experiments were then performed. (A) RT-PCR and (B) quantitative real-time PCR showed that MPT0B169 inhibited *Bcr-Abl* expression. Values are the mean ± SEM from three independent experiments. * *p* < 0.05 versus the untreated control. (C and D) Protein levels of Bcr-Abl and β-actin were determined by Western blotting. Western blots are representative of three experiments, and values are shown as the mean ± SEM. * *p* < 0.05 versus the untreated control. (E) Levels of phospho-Akt, Akt, phospho-ERK1/2, ERK1/2, phospho-STAT3, and STAT3 were determined. β-actin was used as a loading control. RT-PCR and Western blot analyses show one representative result of three independent experiments (A and E).

To investigate the mechanism of the MPT0B169-inhibited cell growth in K562, IMR2, and IMR3 cells, we analyzed the effect of MPT0B169 on the Bcr-Abl expression level and its downstream signaling. Bcr-Abl is required for the proliferation and survival of CML cells. Because treatment with MPT0B169 for 48 h can significantly inhibit cell proliferation (Fig 3B), K562, IMR2, and IMR3 cells were treated with MPT0B169 for 48 h, and Bcr-Abl RT-PCR was performed. MPT0B169 strongly reduced the expression level of Bcr-Abl (Fig 4A). MPT0B169 inhibited the RNA level of Bcr-Abl by approximately 65%–75% in K562, IMR2, and IMR3 cells according to quantitative real-time PCR analysis (Fig 4B). After 48 h of MPT0B169 treatment, the Bcr-Abl protein decreased by 64%–68% in these cells (Fig 4C and 4D). Because MPT0B169 downregulates the *Bcr-Abl* gene, we investigated whether this antitubulin agent interferes with Bcr-Abl downstream signaling pathways Akt, ERK1/2, and STAT3. A Western blot analysis showed that MPT0B169 induced substantial decreases in the total levels and phosphorylation of Akt, ERK1/2, and STAT3 in K562, IMR2, and IMR3 cells (Fig 4E). These results suggest that MPT0B169 inhibited Bcr-Abl expression and its downstream signaling pathways.

### MPT0B169 inhibits tubulin polymerization in K562 and IMR cells

**Fig 5:**
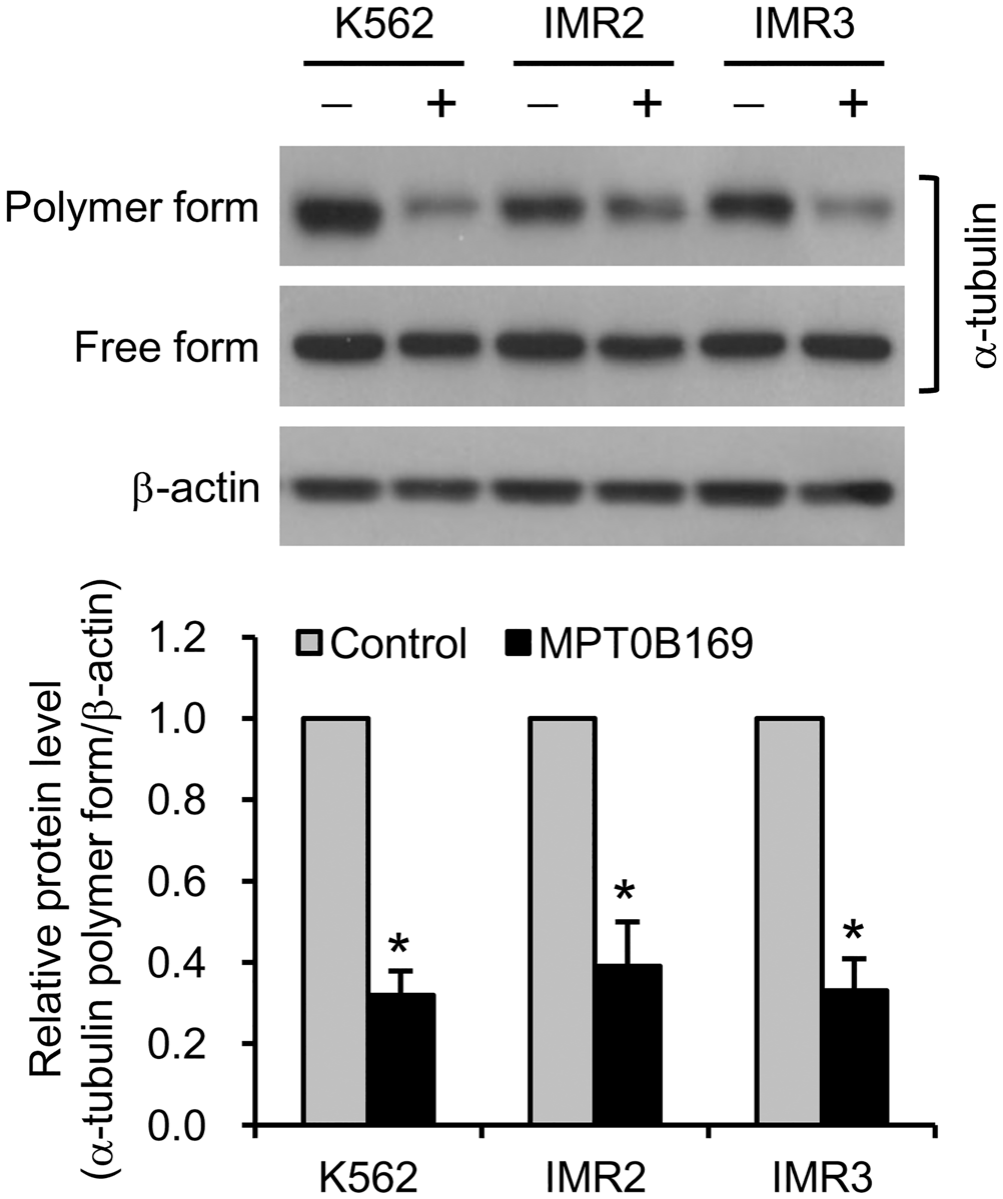
MPT0B169 inhibits tubulin polymerization in K562 and IMR cells. Cells were treated with or without 2.5 μM MPT0B169 for 24 h. The polymer and free form of tubulin were analyzed by Western blot analysis with an anti-α-tubulin antibody. These data show representative results from one of three independent experiments. The bottom panel shows the fold change of the tubulin polymer level in the presence of MPT0B169 relative to the control. Values are the mean ± SEM from three independent experiments. * *p* < 0.05 versus the untreated control.

Although MPT0B169 is a new antitubulin agent [11], we attempted to ascertain whether it inhibits tubulin polymerization in K562, IMR2, and IMR3 cells. Our previous study showed that treatment with MPT0B169 for 24 h inhibited tubulin polymerization in AML cells [11]. We performed an *in vitro* microtubule assembly assay, which respectively separated polymerized and depolymerized microtubules in the pellet and supernatant fractions. Western blot analysis showed that cells treated with MPT0B169 for 24 h exhibited a reduced amount of tubulin polymers, confirming the inhibitory effect on microtubule polymerization (Fig 5).

### MPT0B169 induces G2/M phase arrest and alteration in the protein levels of G2/M regulators

**Fig 6:**
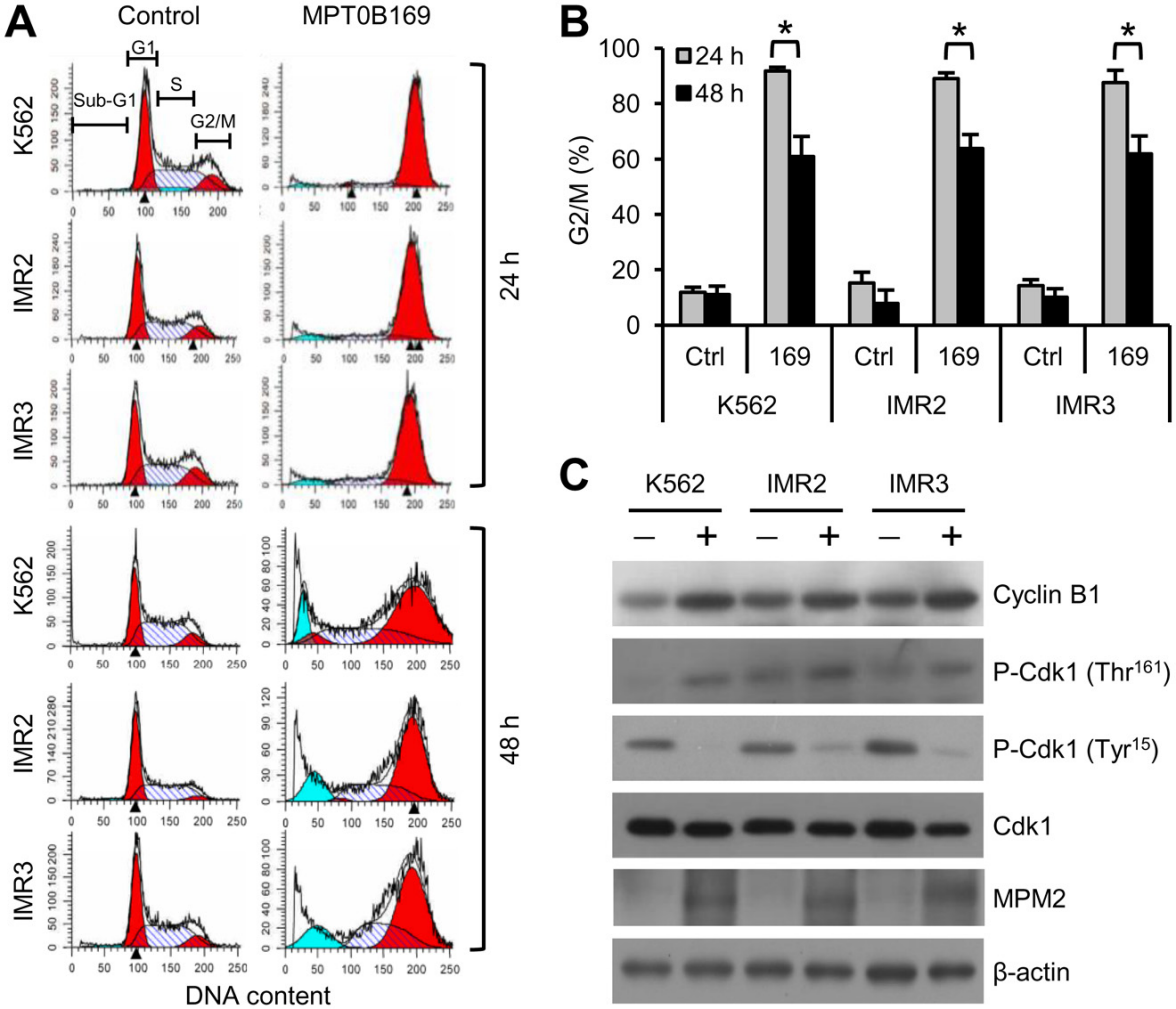
MPT0B169 alters the cell cycle distribution and protein levels of G2/M regulators in K562 and IMR cells. Cells were treated with or without 2.5 μM MPT0B169 for 24 and 48 h, and the following experiments were then conducted. (A) Cell cycle distribution (G1, S, and G2/M) was analyzed by PI staining and flow cytometry. The blue peak indicates sub-G1 cells. These data show representative results from one of three independent experiments. (B) Percentage analysis of the G2/M phase cells shown in (A). Values are the mean ± SEM. * *p* < 0.05. (C) After 24-h treatment, the levels of cyclin B1, phospho-Cdk1 (Thr^161^), phospho-Cdk1 (Tyr^15^), Cdk1, and MPM2 were determined by Western blotting. β-actin was used as a loading control. The figure shows one representative result of three independent experiments.

Because MPT0B169 disrupted microtubule polymerization, we investigated whether this inhibitor affected cell cycle arrest at the G2/M phase. K562, IMR2, and IMR3 cells were treated with MPT0B169 for 24 and 48 h and then analyzed by PI staining and flow cytometry. As the flow cytometry diagrams (Fig 6A) and statistics (Fig 6B) show, MPT0B169 arrested the cell cycle at the G2/M phase in all cell clones after 24- and 48-h treatment. However, a lower percentage of K562, IMR2, and IMR3 cells were arrested at the G2/M phase at 48 h of treatment (approximately 60% arrested) than at 24 h of treatment (approximately 90% arrested) (Fig 6B). In addition, the cells in the sub-G1 population (apoptotic cells) increased at 48 h of treatment (Fig 6B). The cyclin B1 and Cdk1 complex plays crucial roles in regulating the transition from the G2 to M phases [19]. Phosphorylation of the Thr161 site and dephosphorylation of the Tyr15 site are required for Cdk1 activation [20]. We thus further examined the protein levels of cyclin B1 and phospho-Cdk1 in MPT0B169-treated cells. Western blot analysis showed that MPT0B169 increased the protein levels of cyclin B1 and phospho-Cdk1 (Thr161) and reduced the level of phospho-Cdk1 (Tyr15) in K562, IMR2, and IMR3 cells (Fig 6C). We also detected peptides that are phosphorylated only in mitosis by using MPM2 antibody [21]. As shown in Fig 6C, the levels of MPM2 phosphopeptides increased after the treatment of K562, IMR2, and IMR3 cells with MPT0B169 for 24 h. These results indicate that MPT0B169 caused G2/M arrest and changes in the protein levels of cyclin B1 and phospho-Cdk1.

### MPT0B169 induces apoptosis and the mitochondrion-associated apoptotic signaling pathway in K562 and IMR cells

**Fig 7:**
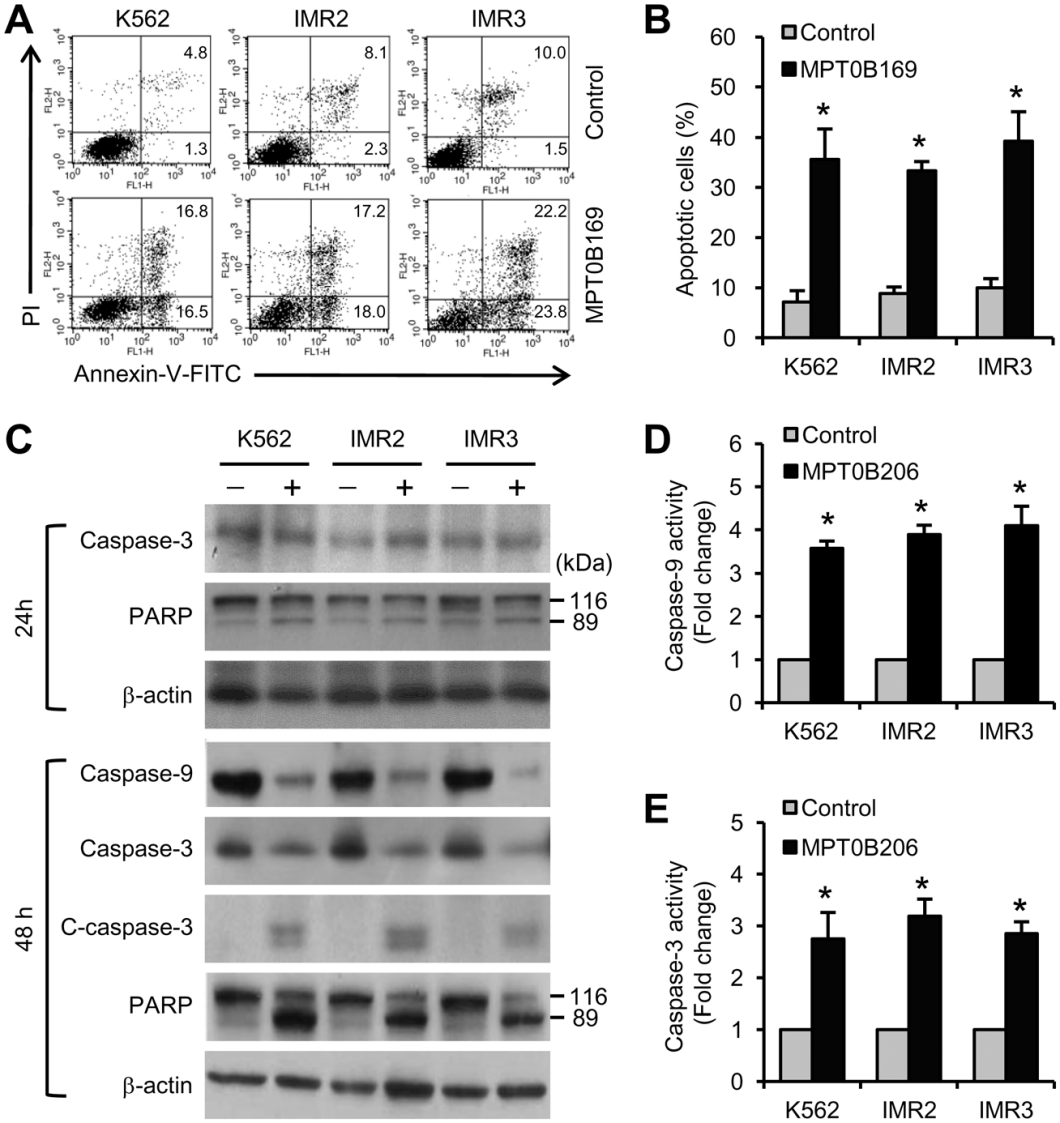
MPT0B169 induces apoptosis and caspase activity in K562 and IMR cells. Cells were treated with or without 2.5 μM MPT0B169 for 24 and 48 h, and the following experiments were then conducted. (A) After 48-h treatment, apoptotic cells were measured by annexin V-FITC/PI staining and flow cytometry. These data show representative results from one of three independent experiments. (B) The percentage of apoptotic cells shown in (A). Values are the mean ± SEM. * *p* < 0.05 versus the untreated control. (C) After 24- and 48-h treatment, the levels of caspase-9, caspase-3, cleaved caspase-3 (C-caspase-3), PARP (full length PARP: 116 kDa; cleaved PARP: 89 kDa), and β-actin were determined by Western blotting. The figure shows one representative result of three independent experiments. (D and E) The activity of caspase-9 and caspase-3 was analyzed in the presence and absence of MPT0B169. Values are the mean ± SEM from three independent experiments. * *p* < 0.05 versus the untreated control.

Subsequently, we analyzed the effect of MPT0B169 on apoptosis in K562, IMR2, and IMR3 cells. Cells were treated with MPT0B169 for 24 and 48 h, and apoptotic cells then were detected by annexin-V/PI staining and flow cytometry. As shown in Fig 7A, treatment with MPT0B169 for 48 h induced an increase in both annexin V^+^ PI^−^and annexin V^+^ PI^+^ cells, suggesting an increase in both early and late apoptosis in K562, IMR2, and IMR3 cells. The statistics showed that MPT0B169 induced significant apoptosis in all cell clones (Fig 7B). Caspase cascades play central roles in apoptotic signals [22]. We thus examined whether the activation of caspases was associated with the apoptotic process induced by MPT0B169. We found that the cleavage of caspase-3 and PARP was not substantially increased compared with the control in all cell clones after 24-h treatment (Fig 7C). However, the protein levels of caspase-9 and caspase-3 notably decreased during treatment with MPT0B169 for 48 h (Fig 7C). In addition, MPT0B169 induced the cleavage of caspase-3 and PARP after 48-h treatment (Fig 7C). A caspase enzymatic activity assay showed that MPT0B169 induced increases in the activity of caspase-9 and caspase-3 in K562, IMR2, and IMR3 cells after 48-h treatment (Fig 7D and 7E).

**Fig 8:**
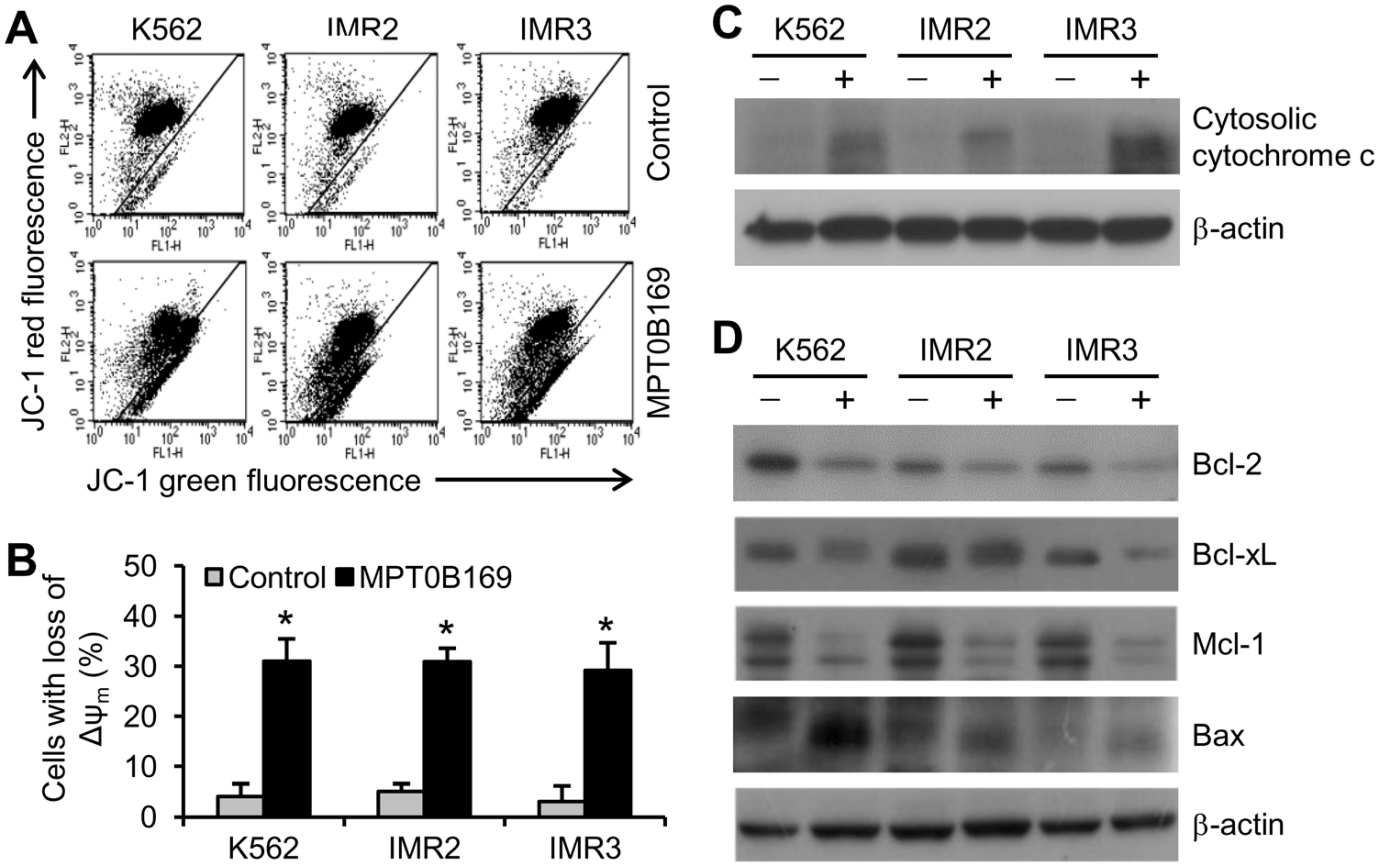
MPT0B169 triggers a loss of mitochondrial membrane potential (Δψ_m_) and alters the level of Bcl-2 family proteins in K562 and IMR cells. Cells were treated with or without 2.5 μM MPT0B169 for 48 h, and the following experiments then were conducted. (A) MPT0B169 induction of the loss of Δψ_m_ was analyzed by flow cytometry after staining cells with JC-1. These data show representative results from one of three independent experiments. (B) Data are presented as the mean ± SEM shown in (A). * *p* < 0.05 versus the untreated control. (C) Cytochrome c in cytosolic fractions was measured by Western blotting. (D) Levels of Bcl-2, Bcl-xL, Mcl-1, Bax, and β-actin were determined by Western blotting. C and D each show one representative result of three independent experiments.

Mitochondria play an essential role in the mechanism of apoptosis [22]; thus, we investigated the effect of MPT0B169 on the mitochondrial membrane potential (ΔΨ_m_). An increase in the loss of mitochondrial membrane potential was observed after treatment with MPT0B169 for 48 h in K562, IMR2, and IMR3 cells (Fig 8A and 8B). Western blot analysis showed that cytochrome c was released into the cytosol [22] in all these cells in response to MPT0B169 (Fig 8C). Subsequently, we examined whether MPT0B169 affected the protein levels of Bcl-2 family proteins. The levels of the antiapoptotic proteins, Bcl-2, Bcl-xL, and Mcl-1, decreased and the Bax protein level increased in MPT0B169-treated cells (Fig 8D). These results suggest that a mitochondrion-associated signaling pathway is involved in MPT0B169-mediated apoptosis in K562 and IMR cells.

## Discussion

In this study, our findings indicate that MPT0B169, a new antitubulin agent, inhibits Bcr-Abl expression and induces cell cycle arrest at the G2/M phase, resulting in growth inhibition and mitochondrion-associated apoptosis in nonresistant and imatinib-resistant CML cells.

Bcr-Abl-dependent [23–25] and -independent [16,18,26] mutations can account for imatinib resistance in CML cells. In the present study, IMR cells exhibited no response to imatinib treatment. This drug resistance was due to neither Bcr-Abl overexpression nor an increase in Bcr-Abl activity. Imatinib could still inhibit phosphorylation of Bcr-Abl. According to these results, we speculate that mutations may not occur in the kinase activity of Bcr-Abl in IMR cells. Sequence analysis of the *Bcr-Abl* gene, especially its kinase domain, can verify this speculation in the future. A Bcr-Abl-independent mutation may be the mechanism for the loss of an imatinib response in IMR cells. To further analyze the cause of resistance, we investigated whether a mutation occurred in the Bcr-Abl downstream pathway. Of note, activation of ERK2 was found to be responsible for imatinib resistance in CML cell lines and a CML patient [16]. In addition, Bonhoure *et al*. reported that ERK1/2 can participate in the mechanism of imatinib resistance in CML cells [27]. In agreement with previous studies, our data showed that ERK1/2 overactivation, especially the hyperphosphorylation of 42-kDa ERK2, was associated with the loss of imatinib sensitivity in IMR2 and IMR3 cells. Overexpression of MDR1 revealed another mode of imatinib resistance in CML cells [18]. MDR1 overexpression was also observed in the mRNA and protein levels in resistant clones. Therefore, resistance to imatinib in IMR2 and IMR3 cells may depend on ERK1/2 overactivation and MDR1 overexpression.

Bcr-Abl activates multiple signal transduction pathways including the Akt, ERK1/2, and STAT3 pathways; these pathways can support CML cell survival and proliferation [2,28]. Activation of these pathways is also critical for the antiapoptotic effect of Bcr-Abl-transformed cells [2,3,28]. Phosphorylation of Akt, ERK1/2, and STAT3 was reported to be an indicator of Bcr-Abl kinase activity [29]. In the current study, MPT0B169 inhibited Bcr-Abl expression. Thus, we examined whether MPT0B169 suppressed Bcr-Abl signal transduction. We found that MPT0B169 substantially inhibited the expression and phosphorylation levels of Bcr-Abl and the phosphorylation levels of its downstream targets, Akt, ERK1/2, and STAT3. Although ERK1/2 phosphorylation markedly increased in IMR2 and IMR3 cells, MPT0B169 could still reduce the phosphorylation levels. These results indicate that MPT0B169 suppresses Bcr-Abl kinase activity and its downstream signaling pathways in nonresistant and imatinib-resistant cells by inhibiting Bcr-Abl expression. In addition, the total protein levels of Akt, ERK1/2, and STAT3 decreased in MPT0B169-treated cells, suggesting that MPT0B169 may affect multiple targets other than Bcr-Abl. Our findings suggest that the inhibition of Bcr-Abl expression is at least one of the major causes of the effect of MPT0B169 on growth inhibition and apoptosis in nonresistant and imatinib-resistant cells. Several studies have suggested that inhibiting Bcr-Abl expression is a promising strategy for overcoming imatinib resistance [30,31].

Our results show that MPT0B169 arrested cell cycle progression at the G2/M phase in nonresistant and imatinib-resistant cells. We therefore investigated the effect of MPT0B169 on changes in the key G2/M regulators, cyclin B1 and Cdk1. Both cyclin B1 and Cdk1 are essential for cells to enter mitosis from the G2 phase [32]. Cdk1 activity is modulated by cyclin B1 binding, the phosphorylation of Thr161, and the dephosphorylation of Tyr15 [20]. Abnormal cyclinB1 accumulation and Cdk1 activity may lead to cell cycle arrest and subsequent apoptosis. MPT0B169 caused the accumulation of cyclin B1 in K562, IMR2, and IMR3 cells. We also observed that phosphorylation at Thr161 (activating site) and dephosphorylation at Tyr15 (inhibitory site) of Cdk1 were induced in MPT0B169-treated cells. These results suggest that MPT0B169 might increase Cdk1 kinase activity and activate the Cdk1/cyclin B1 complex. Furthermore, MPT0B169 treatment increased the expression level of mitosis-specific phosphopeptides identified by MPM2 antibodies [21]. These alterations in protein expression levels are consistent with mitotic arrest [33]. Previous studies have shown that the mitotic arrest of the cell cycle can trigger apoptosis [34,35]. The time of mitotic arrest induced by MPT0B169 was demonstrated to precede that of cell death. In this study, treatment with MPT0B169 for 24 h induced cell cycle arrest at the G2/M phase in almost all cells, but did not induce the caspase-3/PARP apoptotic pathway. Treatment with MPT0B169 for 48 h showed a concomitant decrease in G2/M-arrested cells and an increase in apoptotic cells. Furthermore, we observed early apoptotic cells in addition to late apoptotic cells after 48-h treatment. These results suggest that the cells arrested at the G2/M phase by MPT0B169 eventually underwent apoptotic death.

Intrinsic (mitochondria) and extrinsic (death receptor) apoptotic pathways are two major pathways for inducing apoptosis [22]. Our results showed that MPT0B169 reduced the protein levels of caspase-9 and caspase-3 in nonresistant and imatinib-resistant cells. The cleaved form of caspase-3 and the caspase-3 substrate, PARP, increased in MPT0B169-treated cells, suggesting that MPT0B169 could induce caspase-3 activation. Thus, we further confirmed that MPT0B169 can induce activity of caspase-9 and caspase-3 by using a caspase enzymatic activity assay. Protein levels of Fas and the Fas ligand did not significantly change in response to MPT0B169 (data not shown). These results suggest that MPT0B169 can trigger the caspase-9/caspase-3 intrinsic signaling pathway.

Mitochondria play a critical role in the intrinsic apoptotic pathway [22], and we demonstrated that MPT0B169 caused the loss of mitochondrial membrane potential in treated cells by using a JC-1 staining assay. A change in the mitochondrial membrane potential causes the outer membranes of mitochondria to become permeabilized, and cytochrome c is then released. When cytochrome c enters the cytosol, it binds to caspase-9, forming apoptosomes and eventually activating caspase-3 [36]. Our results showed that MPT0B169 induced cytochrome c release into the cytoplasm. Member proteins of the Bcl-2 family are key regulators of mitochondrion-mediated apoptosis [37]. They include antiapoptotic proteins such as Bcl-2, Bcl-xL, and Mcl-1 and proapoptotic proteins such as Bax [37]. Bax induces cytochrome c release from mitochondria and apoptosis [38,39]. Bcl-2, Bcl-xL, and Mcl-1 promote cell survival through suppressing apoptosis by inhibiting Bax [35,37,40]. Increased protein levels of Bcl-2, Bcl-xL, and Mcl-1 have been suggested to protect cells against apoptosis in CML [41–43]. MPT0B169 reduced the protein levels of Bcl-2, Bcl-xL, and Mcl-1 and increased the Bax level. Therefore, our results suggest that mitochondrion-mediated intrinsic pathways are involved in MPT0B169-induced apoptosis.

In conclusion, this study presents a new tubulin inhibitor, MPT0B169, which inhibits Bcr-Abl expression, arrests the cell cycle at the G2/M phase, and induces mitochondrion-dependent apoptosis in nonresistant and imatinib-resistant cells. MPT0B169 may provide a novel approach for overcoming CML drug resistance and serve as a lead compound for further animal study and drug development.
