# Correction: MPT0B169, a New Antitubulin Agent, Inhibits Bcr-Abl Expression and Induces Mitochondrion-Mediated Apoptosis in Nonresistant and Imatinib-Resistant Chronic Myeloid Leukemia Cells

**DOI:** 10.1371/journal.pone.0186531

**Published:** 2017-10-11

**Authors:** Shuit-Mun Wong, Fu-Hwa Liu, Yueh-Lun Lee, Huei-Mei Huang

The affiliation for the third author is incorrect. Yueh-Lun Lee is affiliated with: Department of Microbiology and Immunology, School of Medicine, College of Medicine, Taipei Medical University, Taipei, Taiwan.
